# Insecticide Resistance Mutations, Enzymatic Activity, and Pathogen Infection in *Culex quinquefasciatus* from Haiti

**DOI:** 10.3390/insects17030331

**Published:** 2026-03-18

**Authors:** Primrose Tanachaiwiwat, Neil D. Sanscrainte, Bernard A. Okech, Alden S. Estep

**Affiliations:** 1 College of Agriculture and Life Sciences, University of Florida, Gainesville, FL 32608, USA; ptanachaiwiwat@ufl.edu; 2 Mosquito & Fly Research Unit, Center for Medical, Agricultural and Veterinary Entomology, Agricultural Research Service, United States Department of Agriculture, 1700 SW 23rd Drive, Gainesville, FL 32608, USA; neil.sanscrainte@usda.gov; 3 Department of Preventive Medicine and Biostatistics, School of Medicine, Uniformed Services University of the Health Sciences, Bethesda, MD 20814, USA

**Keywords:** Haiti, *Culex*, insecticide resistance marker, knockdown resistance (*kdr*), acetylcholinesterase (*AchE*), metabolic resistance, enzymatic resistance, arbovirus, pathogen

## Abstract

Haiti is a Caribbean country of about 11 million people with a high burden of mosquito-transmitted disease and limited vector control, thereby making effective operational mosquito control of high importance. Previous studies have examined vector-borne disease burden and insecticide resistance markers in Haitian *Aedes* and *Anopheles* mosquitoes, but not *Culex* species. In this study, we examined collections of *Culex quinquefasciatus* from 12 locations in northern and southern Haiti for the presence of markers of insecticide resistance (using a variety of target-site mutations and biochemical assays) and pathogens (using a deep-sequencing microbiome workflow). The metagenomic analysis identified *Wolbachia*, *Rhabdoviridae*, and *Plasmodium* infections in all sample pools at relatively high levels, along with less frequent detections of other potential pathogens. Insecticide resistance marker examination identified variable frequencies of knockdown resistance and acetylcholinesterase resistance mutations, as well as variation in resistance-associated enzymatic activities in these populations. These findings indicate that insecticide resistance to pyrethroid and organophosphate insecticides is likely. Although there was variation among *Culex* mosquito populations and no clear activity pattern, enzymatic activity was significantly higher at the southern sites than at the northern sites. Similar findings in *Cx. quinquefasciatus* populations in other locations in the Americas strongly suggest that vector control with pyrethroid and organophosphate adulticides may be of limited efficacy.

## Simple Summary

Haiti has a high burden of mosquito-transmitted diseases and very limited vector control activities. Therefore, effective operational mosquito control is important. Previous studies have examined insecticide resistance in Haitian *Aedes* and *Anopheles* mosquitoes, but not *Culex* species. In this study, we examined collections of *Culex quinquefasciatus* from 12 locations in northern and southern Haiti for markers of insecticide resistance and pathogens. Metagenome analysis identified ubiquitous infection of these *Cx. quinquefasciatus* with symbiotic bacteria, insect-specific viruses, and avian malaria. The presence of target-site insecticide resistance markers and elevated enzymatic activities in these mosquito populations indicates insecticide resistance is likely. We also found that these insecticide resistance markers were generally higher in southern locations near the capital, Port-au-Prince. The findings suggest that *Cx. quinquefasciatus* mosquito control with pyrethroid and organophosphate adulticides may be of limited efficacy.

## 1. Introduction

In Haiti, vector-borne diseases remain endemic largely due to poor infrastructure, a tropical climate, and frequent natural disasters. *Culex quinquefasciatus* is the principal transmitter of Bancroftian filariasis, which is hyperendemic in Haiti and is found in 87.9% of Haitian districts. The disease is a leading cause of permanent disability worldwide, with permanent disfigurement occurring from lymphedema in the limbs [1,2]. *Culex quinquefasciatus* is also a competent vector for arboviruses such as West Nile virus (WNV) [3,4], St. Louis encephalitis virus [5,6,7], and possibly Rift Valley Fever [8]. West Nile virus—carried by *Cx. quinquefasciatus* and other members of the *Cx. pipiens* complex—appeared in 2/116 patients in a study of mosquito-borne diseases in Gonaïves, Haiti. However, there is speculation that false-positive commercial ELISA tests may frequently misdiagnose WNV as dengue, thus obscuring the actual prevalence of the disease [9,10]. In general, WNV is the most prevalent arboviral disease in North America and is endemic in several regions of Africa and Asia [11]. The *Cx. pipiens* complex is involved in the transmission of other pathogens, including avian malaria and avian pox virus. Although *Cx. pipiens* is primarily ornithophilic, it plays a role in the mosquito–bird amplification cycle of WNV that can transmit to “dead-end” hosts such as humans [12]. The transmission of WNV by *Culex* species has resulted in 40,000 clinical cases and almost 1700 human deaths in the United States alone [12,13]. Although Haiti currently has a low prevalence of WNV, its initial presence in Hispaniola was likely due to migratory birds from the Americas [10].

*Culex* species are difficult to distinguish morphologically; larval forms lack distinguishing characteristics, and species within the *Cx. pipiens* complex (such as *Cx. quinquefasciatus)* are morphologically alike. Additionally, members of this complex are known to hybridize, further complicating identification between species [14]. It is unclear how this hybridization affects vector competence for diseases such as lymphatic filariasis and WNV. In general, this indistinctness contributes to the lack of precise targeting in vector control. Compared with *Aedes* and *Anopheles*, *Culex* species have not been targeted as aggressively by insecticides, nor has *Culex* insecticide resistance been comprehensively studied. *Culex* and *Aedes* species tend to live near one another—particularly *Ae. aegypti* and *Cx. quinquefasciatus*, which are both domestic species adapted to urban environments and human cohabitation. Off-target exposure from vector control programs targeted towards *Aedes* and *Anopheles* has allowed several mechanisms of resistance to pyrethroids and organophosphates to evolve in *Culex* species. The two major mechanisms of resistance to pyrethroids in *Culex* are mutations in voltage-sensitive sodium channels that cause target-site insensitivity and overexpression of cytochrome P450(s) that increase detoxification. Voltage-sensitive sodium channel mutations occurring at L1014F are termed knockdown resistance (*kdr*) and are generally incompletely recessive in *Culex* [12]. Additionally, a single nucleotide polymorphism (SNP) in the acetylcholinesterase gene (*AchE*), termed G119S, has been significantly associated with organophosphate resistance [15,16]. Long-term use of insecticides can lead to the development of insecticide resistance, posing significant public health concerns. In countries intensively using pyrethroids to reduce dengue impact, *kdr* mutations and significant levels of insecticide resistance were observed in *Culex*, an off-target species in the vector control program. In countries where *Culex* frequently carries filariasis or WNV, this off-target resistance is a blind spot in vector control programs that may result in increased spread of such vector-borne diseases [17].

Previous research on insecticide resistance in Haiti focused on *Ae. aegypti* and found phenotypic expression of pyrethroid resistance in field assays that were not reproducible in the lab, observing the *kdr* allele in very few samples [18]. However, in a study performed seven years later, 70% of the Leogane and Merger population displayed allelic *kdr* mutations, indicating a rapid and widespread increase in pyrethroid resistance [19]. In the French West Indies, Brazil, Nigeria, and Cameroon, the widespread use of insecticides against *Aedes* led to off-target reduced susceptibility in *Culex* populations [16,20,21,22]. It is reasonable to postulate that an increase in *kdr* mutations in Haitian *Aedes* populations is matched by a proportionate increase in mutations in the *Culex* populations. The G119S mutation is present in populations of *Culex* in the Caribbean and on the island of Guadeloupe in the French West Indies, with an incidence higher than that of the wildtype “susceptible” allele due to vector control of *Ae. aegypti* [20]. However, we could not find research studies assessing the levels of organophosphate resistance in *Culex* within Haiti specifically.

Though Haiti has programs in place to reduce the impact of vector-borne diseases (in particular, lymphatic filariasis), these programs primarily focus on long-term treatment rather than prevention. The efficacy of mosquito vector control in reducing the spread of vector-borne disease is understudied in Haiti, though previous research has shown that pyrethroid-treated bed nets are effective [23]. There is a lack of existing research delineating the impact of vector-control-based disease reduction or the prevalence of pathogens carried by *Culex* species in Haiti. Additionally, research assessing *Culex* insecticide resistance is lacking and would be useful for guiding further mosquito vector control directives in Haiti. This study partially addresses this gap by providing baseline information from samples collected from 12 locations in Haiti during 2017. This study assessed the frequency of target-site mutations and quantified the enzymatic activities of esterases, cytochrome P450s, and glutathione S-transferases linked to insecticide resistance in *Culex*. We also assessed pathogen presence in these *Cx. quinquefasciatus* by conducting a microbiome study.

## 2. Materials and Methods

### 2.1. Arthropod Surveillance Collection Procedures

**Figure 1:**
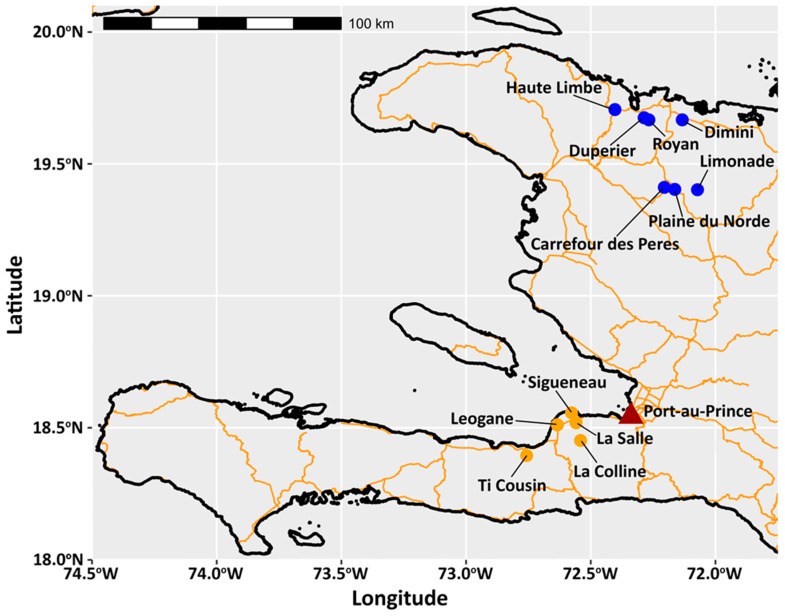
Sampling locations. Black outlines represent the coastline of Haiti. Orange lines represent primary and secondary roads. Filled circles represent sampling locations in Northern (blue) and Southern (orange) Haiti. The capital, Port-au-Prince, is marked with a red triangle. Figure created in R version 4.4.1 [24].

Mosquitoes were collected from March 2017 to July 2018 using CDC light traps with incandescent bulbs and CDC gravid traps with hay infusion (J.W. Hock, Gainesville, FL, USA), both deployed for 12 h a night, 3 days a week. The 12 locations sampled include seven in northern Haiti, in the Nord department, and five in southern Haiti, west of Port-au-Prince, in the Ouest department (Figure 1). Collection periods varied by location and were limited to 1–2 months. Trapping was conducted with property owner approval. Sampling locations and trapping metadata are listed in File S1.

*Culex quinquefasciatus* complex mosquitoes were retrieved from the trap nets, identified based on morphology, and transferred to microcentrifuge tubes and then stored frozen at −80 °C at the University of Florida Public Health Field Laboratory in Gressier, Haiti, until shipment to the Emerging Pathogens Institute at the University of Florida in Gainesville, FL, for subsequent sample preparation (Section 2.3).

### 2.2. Control Strain

*Culex quinquefasciatus* has been established at the Center for Medical, Agricultural, and Veterinary Entomology (CMAVE), USDA-ARS, in Gainesville, FL, since 1995, from an Orlando, FL, strain. Mosquitoes were reared using a standard procedure wherein collected egg rafts were reared in 3 L plastic trays [25,26,27]. One-half gram of larval diet (2:1 alfalfa powder/pig chow) was added to each tray every other day. Pupae were collected approximately 1 week after placing eggs in water, transferred to a screened cage, and maintained at 27 °C and 80% RH. Incandescent lighting simulated a crepuscular profile with a 14 h/10 h (L/D) photoperiod. Adults were fed 10% sucrose ad libitum and manually defibrinated bovine blood, which was warmed to 37 °C and provided twice weekly [27].

### 2.3. Sample Homogenization, Pooling, and RNA Purification

Forty individual females collected from each of the different locations (480 total individuals) (File S1) were loaded into wells of 96-deep well plates (Omni International, Kennesaw, GA, USA) in 400 µL of 100 mM sodium phosphate, pH 7.4, with 2.0 mm zirconia beads. The plates were sealed with Teflon sealing mats and homogenized for 60 s at 30 hertz. The samples were centrifuged for 2 min at 805× *g* and then refrozen until enzyme assay and RNA preparation [13].

Individual samples were assayed for enzyme activity directly from the sodium phosphate homogenate as described below (Section 2.5). Homogenized samples were diluted 1:5 in nuclease-free water (NFW) for *kdr* and *AchE* target site mutation assays, as well as the *Culex* speciation assay (Section 2.4).

Samples for RNA purification and sequencing (Section 2.6) were produced by combining 20 µL of homogenate from eight individuals at the same location (a single column on the 96-well plate) into a single pool. Thus, five pooled samples were generated from each of the 12 locations. RNA was purified from each pool using a commercial silica spin column kit (Zymo Research, Irvine, CA, USA) after dilution with DNA-binding buffer at a 2:1 ratio. Purification followed the manufacturer’s instructions, and elution was performed with 20 µL of NFW applied directly to the column matrix.

Complementary DNA was generated using the SuperScript IV kit (Thermo Fisher Scientific, Waltham, MA, USA). Briefly, 1 µL of 50 ng/µL random hexamers, 1 µL of 10 mM dNTP, 11 µL of generated template RNA, and 2 µL of NFW were combined to a final volume of 15 µL. Samples were heated to 65 °C for 2 min and then placed on ice to reduce secondary structure. A reverse transcription reaction comprising 4 µL of 5× SSIV Buffer, 1 µL 100 mM DTT, 1 µL ribonuclease inhibitor, and 1 µL SuperScript IV Reverse Transcriptase was added to each sample. After mixing, the samples were incubated at 23 °C for 10 min, then at 50–55 °C for 10 min. The reaction was inactivated by incubation at 80 °C for 10 min. Second-strand synthesis was conducted using the NEBNext Non-directional Second Strand Kit (New England Biolabs, Ipswich, MA, USA).

### 2.4. Speciation, Knockdown Resistance, and Acetylcholinesterase Mutation Detection Assays

Knockdown resistance assays assessed the SNP changing 1014L to 1014F (occasionally serine, position based on the standard *Musca domestica* sodium channel). A melt curve assay (MCA) was performed to identify the presence of the SNP via a difference in melting temperature (Tm). PCR reactions were assembled in 384-well plates on an Eppendorf 5750 workstation (Eppendorf, Hamburg, Germany). Primers and primer concentrations are listed in Table 1. Each 10 μL PCR reaction consisted of 9 µL of master mix containing 5 μL of SYBR Select (Thermo Fisher Scientific, Waltham, MA, USA), primer concentrations as listed in Table 1, and the remainder NFW. One microliter of diluted mosquito homogenate was transferred from the sample plate to the assay plate by the workstation. Reactions were cycled on a QuantStudio6 Flex (Thermo Fisher Scientific, Waltham, MA, USA) for 40 cycles using standard FAST parameters with a final melt curve phase from 60 °C to 95 °C. The presence of *kdr* was assessed by examining individual melt curves to determine T_m_ peaks (~82.2 ± 0.4 °C for 1014F, ~86.0 ± 0.4 °C for 1014L). Heterozygosity at position 1014 was identified by the presence of a peak at both T_m_s [13,28,29].

Detection of the canonical G119S *AchE* SNP described by Weill [15] was conducted using novel MCA primers and the method described above. Primers and primer concentrations are listed in Table 1. Identification of the 119S homozygous mutant (SS, in abbreviated notation) results in an ascending temperature curve with a primary T_m_ peak at 85.8 ± 0.4 °C. The 119G homozygous wildtype (GG) produces a primary T_m_ peak at 80 ± 0.4 °C and a weak shoulder rather than a peak at 85.8 ± 0.4 °C. The G119S heterozygote (GS) produces a primary T_m_ peak at 80 ± 0.4 °C and a distinct T_m_ peak, rather than a shoulder, at 85.8 ± 0.4 °C.

Insecticide resistance SNPs are undetermined in many *Culex* species and *Cx. nigripalpus* are often collected with *Cx. pipiens* complex mosquitoes. Therefore, we included a speciation assay to ensure that the genotyping results for the insecticide resistance MCAs were specific to *Cx. quinquefasciatus*. The separation of *Cx. pipiens/quinquefasciatus* complex mosquitoes from *Cx. nigripalpus* was achieved by MCA with novel primers targeting a cytochrome oxidase I gene region, following the method above. Primer sequences and concentrations are in Table 1. *Culex pipiens* complex mosquitoes result in a distinct T_m_ peak at 84.9 ± 0.2 °C, and *Cx. nigripalpus* results in a distinct T_m_ peak at 70.3 ± 0.2 °C.

All MCAs were performed on the same homogenate plates, with controls appropriate for each assay. This included controls for each allele or species, as well as a DNA-negative control.

### 2.5. Metabolic Resistance Assays

Metabolic assays were conducted on the sodium phosphate buffered homogenates. Each population consisted of 40 individuals, and two populations were assessed per plate. Technical duplicate reactions were conducted for each sample. Conversion of the resulting absorbance values to the appropriate units (mg of protein, micrograms of esterase, etc.) was performed by comparing them to standard curves included on each plate using the Multiscan Sky software version 5.0 (Thermo Fisher Scientific, Waltham, MA, USA).

#### 2.5.1. Bradford Protein Assay

A total protein assay (Bradford Assay) was performed using the MAN0011181 protocol from Thermo Fisher Scientific (Waltham, MA, USA). In total, 100 µL of Coomassie reagent was added to each well, and 10 µL of supernatant was transferred and mixed by pipetting 10 times. A standard curve of BSA dilutions was included on each plate. Plates were scanned on a Multiscan Sky at 595 nm after 15 min of incubation.

#### 2.5.2. Cytochrome P450 Assay

One hundred microliters of 3,3′,5,5′-tetramethylbenzidine (TMBZ) sodium acetate buffer was added to each well. Ten microliters of supernatant was transferred from the homogenate plate into duplicate test wells. Ten microliters of each standard was transferred to two columns (11–12). Then 12.5 µL of 3% H_2_O_2_ was added to each well and mixed 15 times. Plates were incubated for 10 min at room temperature in the dark and scanned on a Multiscan Sky at 620 nm.

#### 2.5.3. Glutathione S-Transferase (GST) Activity Assay

A 1-chloro-2,4-dinitrobenzene (CDNB)–reduced glutathione (GSH) solution was prepared immediately before use by combining 0.51 mL of 21 mM CDNB in methanol with 10.2 mL of 10 mM GSH in 0.1 M sodium phosphate buffer, pH 6.5. After briefly vortexing, 90 µL of the CDNB-GSH solution was added to each well. Ten microliters of 0.1 M sodium phosphate buffer, pH 7.4, was added to columns 11–12 as negative controls, and 10 µL of supernatant was transferred to duplicate columns. The plate was immediately kinetically read on the Multiscan Sky for 5 min at 1-min intervals at 340 nm.

#### 2.5.4. α-Carboxylesterase Activity Assay

Ninety microliters of 30 mM α-naphthyl acetate buffer and 8 μL of sodium phosphate buffer, pH 7.4, were loaded into each well of columns 1–10. One hundred microliters of an α-naphthol dilution series was added to columns 11–12 as a standard curve. After a 15-minute incubation in the dark, 2 μL of supernatant from column 6 was added to columns 1–2 (see Bradford Assay), then the mixture was mixed 15 times. Thirty microliters of Fast Blue B was added to columns 1–4, and the mixture was stirred 15 times. Fast Blue B addition and mixing continued four columns at a time. After a 5-minute incubation in the dark, the plate was read at 600 nm and converted to micrograms based on the standard curve. Results were multiplied by 5× to account for the dilution factor.

#### 2.5.5. β-Carboxylesterase Activity Assay

β-naphthyl acetate assays were conducted as described above, but with β-naphthyl acetate buffer and β-naphthol as the known standard. The β-naphthyl acetate assay wells were read at 550 nm, and results were multiplied by 5× to account for the dilution factor.

### 2.6. Nanopore Sequencing and Bioinformatics

Double-stranded cDNA (dscDNA) pooled samples (5 pooled samples × 12 locations = 60 sequencing libraries) were sequenced on a MinION Mk1B device using the SQK-NBD114.96 ligation sequencing kit and R10 chemistry (Oxford Nanopore Technologies, Oxford, UK) following the manufacturer-provided protocol (version NBE_9171_v114_revP_15Sep2022). Variations from the manufacturer’s protocol at the initial end preparation step were as follows: a reduced quantity of DNA Control Sequence (from 1.0 µL to 0.25 µL per reaction), a reduced input DNA quantity (10 µL of dscDNA, down from 11 µL), and the addition of 1 µL of NFW. Due to dscDNA length bias toward shorter fragments (generally less than 1 kb), we followed the protocol recommendation and loaded 132.6 femtomoles of the final DNA library into the cell. DNA concentrations were determined by Nanodrop 8000 (Thermo Fisher Scientific, Waltham, MA, USA). Sequencing and devices were managed by MinKNOW software version 24.02.16. The initial quality threshold was set to 10, and initial barcode binning was performed in the software.

Raw barcoded sequence data were subsequently filtered a second time to reduce misbinning by requiring a minimum 37-base barcode match at each end and an identity of 95% to the true barcode sequence. Filtered reads were subsequently used for microbiome assessment using the Chan–Zuckerberg ID platform version 8 (czid.org). The resulting assignment files for each sample were screened, and a frequency heatmap was constructed in R using the pheatmap package [30].

## 3. Results

### 3.1. Assessment of Knockdown and Acetylcholinesterase Target Site Mutations

**Figure 2:**
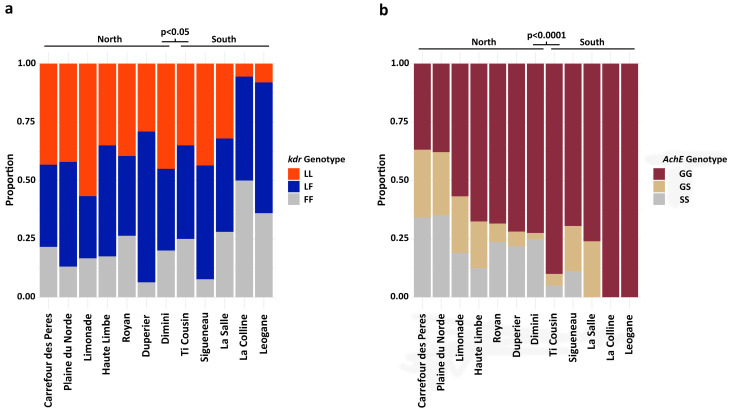
Assessment of target site resistance genotypes in Haitian *Culex quinquefasciatus* populations. (**a**) Genotypes for the L1014F pyrethroid resistance-associated SNP differed between northern and southern Haiti (Χ^2^ = 6.0613, df = 2, *p* = 0.048). (**b**) The G119S organophosphate resistance-associated SNP also differed between northern and southern Haiti (Χ^2^ = 32.819, df = 2, *p*-value = 7.472 × 10^−8^). Both mutations were characterized using a melt curve analysis assay, which included positive controls for each homozygous genotype and the heterozygous genotype.

Melt curve assay testing identified the characteristic *Cx. quinquefasciatus* L1014F mutation in all 12 populations (Figure 2a). Both heterozygotes and homozygotes with the 1014F allele were detected, though the level varied among populations. Duperier and Sigueneau had less than 10% of the 1014FF heterozygote, while La Colline had the most at nearly 50%. Approximately a third of each population was heterozygous for the L1014F SNP, though the level varied. Overall, the frequencies of the 1014F SNP-containing genotypes were significantly elevated in southern versus northern locations (Χ^2^ = 6.0613, df = 2, *p* = 0.048).

Assessment of these same organisms for the G119S *AchE* mutation, a marker of organophosphate insecticide resistance [15], indicated it was also present in both northern and southern Haiti. However, unlike the *kdr* mutation, it was not present in every population (Figure 2b). All three G119S genotypes (GG, GS, SS) were present in all seven northern populations, with the wildtype GG as the predominant genotype. The heterozygous *AchE* mutant SS genotype varied from about 5% in Ti Cousin to ~30% in Carrefour des Peres and Plaine du Nord. In contrast to the northern populations, the SS genotype was absent from three southern populations (La Salle, La Colline, Leogane) and lower in Sigueneau and Ti Cousin than in any of the Northern populations. The heterozygous GS genotype was also absent from La Colline and Leogane. Statistical analysis showed that *AchE* SNP-containing genotypes were significantly higher in northern Haiti than in southern Haiti (Χ^2^ = 32.819, df = 2, *p*-value = 7.472 × 10^−8^).

### 3.2. Metabolic Resistance Assays

Biochemical assays assessing common resistance associated with enzymatic activity also indicated significant differences between northern and southern populations. This includes cytochrome C oxidase (CytC) activity (H(1) = 49.6, *p* = 1.87 × 10^−12^), glutathione-S-transferase (H(1) = 51.9, *p* = 5.87 × 10^−13^), α-esterase (H(1) = 13.2, *p* = 2.75 × 10^−4^) and β-esterase activity (H(1) = 13.9, *p* = 1.95 × 10^−4^).

**Figure 3:**
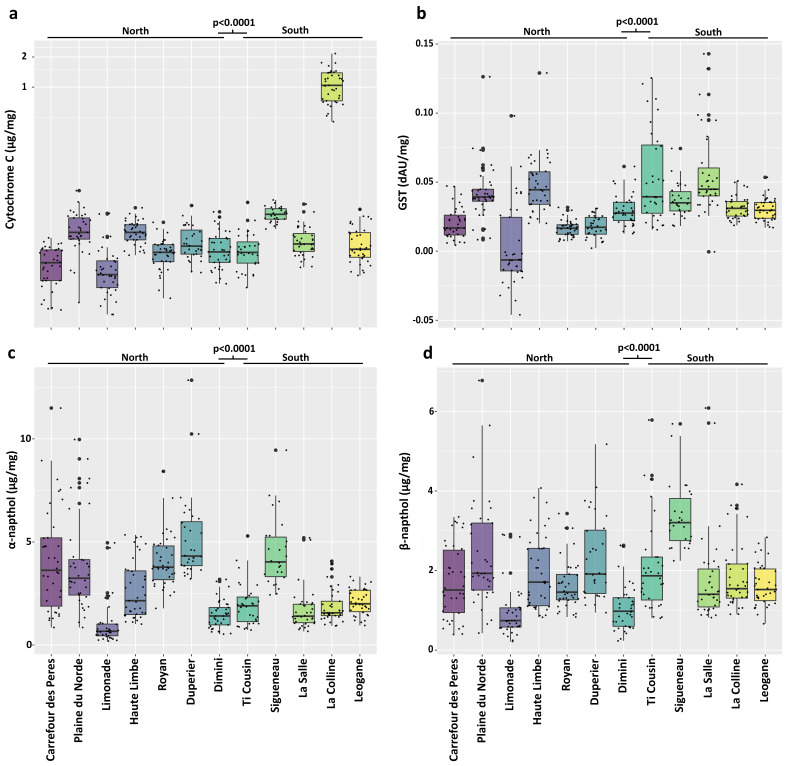
Normalized enzyme activities of 12 Haitian *Culex quinquefasciatus* populations indicate significant differences between grouped northern and southern locations. (**a**) Cytochrome C oxidase (H(1) = 49.6, *p* = 1.87 × 10^−12^), (**b**) Glutathione-S-transferase (H(1) = 51.9, *p* = 5.87 × 10^−13^), (**c**) α-esterase (H(1) = 13.2, *p* = 2.75 × 10^−4^), (**d**) β-esterase (H(1) = 13.9, *p* = 1.95 × 10^−4^). Subsequent application of Dunn’s test for means comparison indicated many significant differences between individual populations. Results of individual means comparisons are included in File S2. The small points represent the individual values summarized in the colored box-and-whisker plot. Points in bold are those that are outside the confidence interval.

At a more granular level, many significant differences between individual populations were identified using means comparisons (Figure 3 and File S2). Cytochrome C activity was significantly higher in the La Colline population than in the other 11 populations, except for Sigueneau, another southern population (Figure 3a). While there were some other significant differences between populations, the remaining populations were much more closely grouped.

Glutathione-S-transferase activity patterns were less clear (Figure 3b). Large interquartile ranges were observed in Limonade, Ti Cousin, La Salle, and Haute Limbe, while narrower variation was observed in the other locations. Notably, despite the broad heterogeneity in the Limonade population, it had significantly lower activity than many other populations (File S2). Other significant differences were found between populations, but no clear pattern emerged (File S2).

While α-esterase activity was significantly elevated in northern populations compared to southern populations, this was not true at the individual population level (Figure 3c). Five of the six highest levels of α-esterase activity were in northern populations, and four of the six lowest α-esterase activity levels were in southern populations. Among the northern populations, Limonade and Dimini had low activity levels, as did most of the southern populations. However, the southern Sigueaneau population had one of the highest α-esterase activities and was significantly higher than those of every other southern population. The overall pattern of β-esterase activity was similar to that of α-esterase, but with greater within-population variability, as indicated by the larger interquartile distances (Figure 3d).

**Figure 4:**
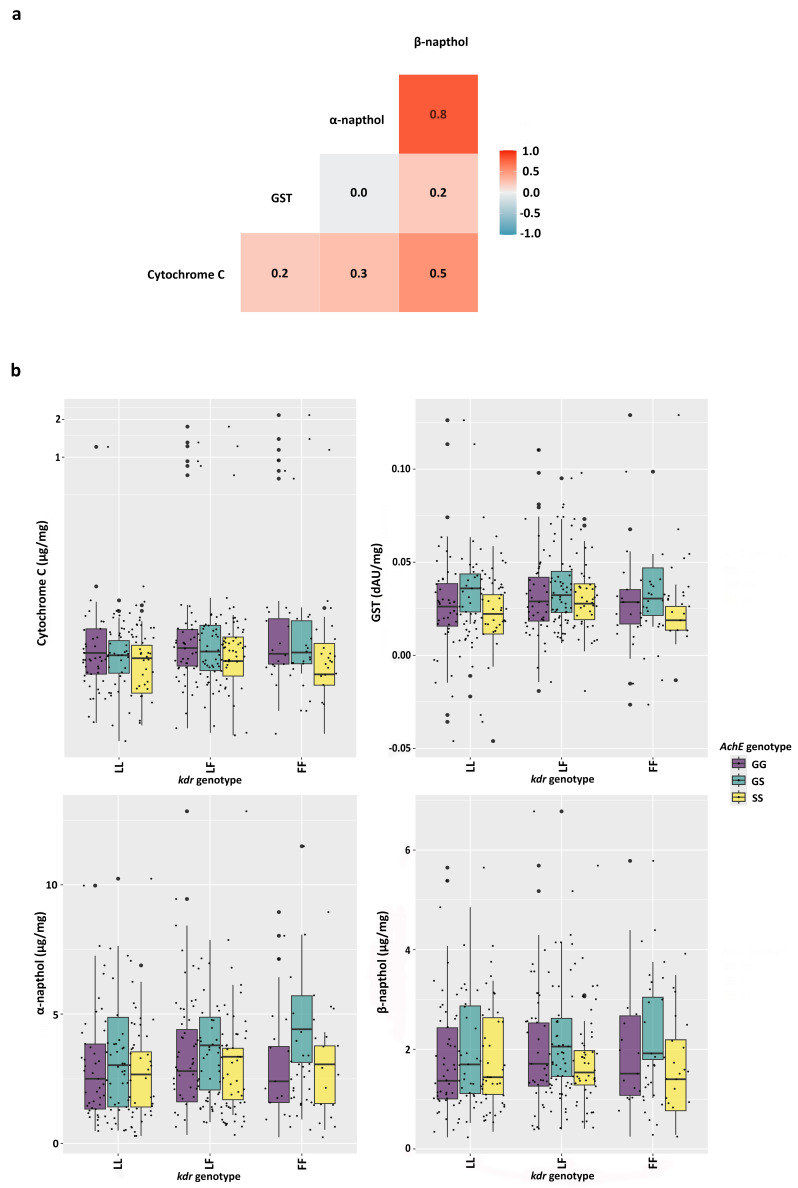
Interactions between resistance mechanisms. (**a**) Spearman correlation analysis of enzyme activities indicates a strong correlation (0.8) between α- and β-esterase activities and a moderate strength correlation between β-esterase and glutathione-S-transferase activity. (**b**) Enzymatic activity did not show a strong bias to any target site resistance mutation.

Having observed a similar pattern between α- and β-esterase activity in these populations, we examined the correlations between enzyme activities using Spearman’s correlation rather than Pearson’s, since we did not know whether the relationships between the variables were linear (Figure 4a). This analysis confirmed there is a very strong correlation (Spearman’s ρ = 0.8) between α- and β-esterase activity. We also detected moderate correlations between α- and β-esterase and CytC activity (α: CytC, ρ = 0.3; β: CytC, ρ = 0.5). All other correlations between enzyme activities were weak (ρ < 0.3).

We also examined the relationship between *kdr* and *AchE* target-site genotypes and enzymatic activity (Figure 4b). We did not observe large differences associated with these target-site resistance genotypes across the four enzyme activities. However, numerically, mosquitoes homozygous for both the *kdr* (FF) and *AchE* (SS) target-site mutations had among the lowest activity levels across all four enzyme families. They had the lowest mean CytC and GST activity, tied for the lowest mean β-esterase activity, and one of the lowest mean α-esterase activities among the genotype combinations.

### 3.3. Microbiome Analysis

**Figure 5:**
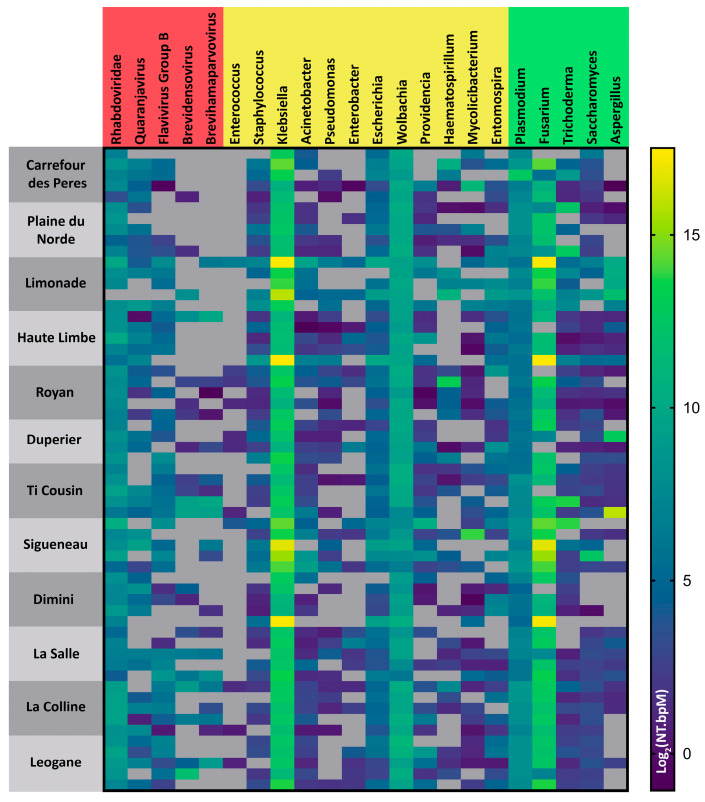
Microbiome abundance in Haitian *Culex quinquefasciatus*. Reads were assigned using the Chan–Zuckerberg identification platform v.8 (czid.org) and expressed as log values of the abundance of NCBI NT database matches per million bases sequenced (NT.bpM). Each line represents an individual sample from the given location. Viral components are in red, bacterial components in yellow, and eukaryotic components in green. Gray tiles indicate zero abundance. Heatmap constructed in R 4.4.1.

Reverse-transcribed RNA was used to examine the microbiomes in these *Culex* samples (Figure 5). Using the Chan–Zuckerberg ID platform, we assigned taxonomic identities to quality-filtered reads for individuals. These assignments and alignment lengths were then used to calculate a normalized frequency for each taxonomic category (NT.bpm = NT/NR database-matching bases per million) to account for individual library-to-library variation in the number of reads. Reads were assigned to genus level, and when possible, to species. Fifty-nine of 60 libraries produced adequate output for analysis. Thus, each location is represented by 5 libraries, except for Duperier, which is represented by 4. The full output from the Chan–Zuckerberg ID platform is provided in File S3.

A few notable commonalities were observed. High frequency of bacterial reads from the *Klebsiella* and *Wolbachia* genera was identified in all libraries. The highest frequencies of eukaryotic reads were identified from the fungal *Fusarium* genus found in 54/59 libraries. The next most frequent eukaryotic reads were from the *Plasmodium* genus, and these were identified in every library. Viral Rhabdoviridae reads were present in 58/59 libraries.

As noted, *Klebsiella*-associated reads were common, but the genera of the other “ESKAPE bacteria” (known for their human pathogenic effects) were much more variable. Reads from the *Escherichia*, *Acinetobacter,* and *Staphylococcus* genera were common and each was identified in more than 75% of the libraries. The other ESKAPE bacterial genera were less common, with *Enterococcus* and *Enterobacter* reads being absent in many libraries. Species-level examination of the generic classifications indicated that the reads from these families were overwhelmingly not specific pathogenic bacterial species.

Notably, in addition to the Rhabdoviridae reads mentioned above, *Quaranjavirus* and *Flavivirus* Group B reads were common in more than half of the libraries, but these were generally insect-associated rather than human-pathogenic. We did not detect reads supporting West Nile virus infection in these samples.

## 4. Discussion

The objectives of this study were two-fold: to examine insecticide resistance-associated markers in Haitian *Culex* mosquitoes and to examine these same mosquitoes for the presence of potential pathogens. Both objectives provide useful data that can serve as a historical baseline for more recent collections, help optimize mosquito control efforts, and assist in assessing mosquito-borne disease risk. As noted above, there is very limited information on insecticide resistance in Haitian mosquitoes, and most studies focus on *Aedes* and anopheline species due to the greater immediacy of diseases such as dengue and malaria. We found no previous studies examining insecticide resistance in *Culex* mosquitoes in Haiti, even though evidence of active *Culex*-transmitted disease is clear. Hispaniola is the only Caribbean island with lymphatic filariasis, accounting for about 90% of cases in the region. Previous studies have reported relatively high infection rates with lymphatic filariasis in *Culex* pools from several locations in Haiti [31]. Haiti also has had two confirmed human WNV cases and relatively high rates of WNV infections of birds on the island. Thus, this remains problematic in Hispaniola [9,10].

With respect to the first objective, we show in Haitian *Cx. quinquefasciatus* that both the L1014F *kdr* and the G119S *AchE* insecticide resistance markers are present. The L1014F and G119S genotypes in our mosquito samples varied significantly between the north and south, with the mutant *kdr* and *AchE* genotypes more frequent in northern Haiti. While at least some 1014F was detected at every site, in a couple of southern sites west of the capital, no 119S alleles were detected. One possible explanation is that northern Haiti is more frequently visited by tourists, particularly around the cruise destination Labadee. Tourists visiting Haiti are advised by the CDC to treat their clothes with permethrin [32,33]. Additionally, the distribution of sample sites excluded Port-au-Prince in the southern region, where levels of resistance markers from tourism or more frequent control interventions may have a greater impact.

The detection of target-site insecticide resistance SNPs, while novel for Haiti, is not unexpected in the context of the existing *Culex* literature, as these mutants are widely found in the Americas and worldwide [13,15]. Recent studies show similar variation in L1014F frequencies among populations [29]. It is notable that the insecticide resistance response in *Cx. quinquefasciatus* is complex, and that phenotypic pyrethroid insecticide resistance is often present even when *kdr* mutants are infrequent [28]. The distribution of the G119S *AchE* mutation is much less explored, but it was first identified and characterized by Weill [15] in the early 2000s in the Caribbean, and little work has been conducted in the Americas since the initial description. G119S is likely found worldwide [34,35,36,37]; hence, our detection in Haiti is not surprising. However, without a substantial body of existing frequency studies for comparison, we cannot say whether the levels identified in Haiti differ from those throughout the Caribbean.

Our enzymatic assay indicated significant differences in CytC, GST, α-esterase, and β-esterase between northern and southern Haiti. However, comparisons of the individual populations revealed many significant differences across all four enzymatic activities measured within both the northern and southern groupings. There were two major findings in these assays. First, CytC activity was significantly higher in the La Colline population than in the others, but the reason for this difference is unknown and was not further explored. Second, there was no clear pattern of enzymatic activity among these populations that would serve as a good marker for resistance.

We found several interesting relationships between enzymatic activities. First, there was a strong correlation between α- and β-esterase activities (Figure 4a). Although additional area-wide studies are needed to confirm this finding beyond Haiti, if this is generally true, it would suggest that assessing both esterase activities is duplicative and that eliminating one assay could be an efficiency-saving measure to reduce labor and cost in insecticide resistance assessment. We also noted a moderate correlation between β-esterase and CytC activity, but a low correlation between other enzyme activity combinations. We also observed that the presence or absence of target-site insecticide resistance mutations did not affect enzymatic activity, suggesting that these mechanisms are uncorrelated.

Additionally, although there are currently no studies on *Plasmodium relictum* (avian malaria) in Haiti, the presence and frequency of *Plasmodium* observed here suggest that greater insight into its epidemiology may be needed. Further analysis to identify what species of *Plasmodium* primarily colonizes Haitian *Cx. qiunquefasciatus* is needed, though it is unlikely to be *P. falciparum*, due to its inability to release sporozoites in this species [38]. The lack of WNV detection aligns with recent studies reporting a low seroprevalence of WNV antibodies (~1%) in the Haitian population. Resident birds are known to carry WNV antibodies and may act as viral reservoirs, suggesting that, despite the low number of reported cases, the confirmed presence of the virus among intermediate hosts still indicates a risk of transmission in the region [39]. Our collection method, using light traps, may also not be optimal for WNV detection [40].

The development of predictive ability is the purpose of assessing markers generally, and this practice has become more common in human medicine as the links between genetics and outcomes become more formalized [41,42,43,44]. The use of high-value markers allows assessment of an easily determined surrogate rather than the direct effect, which can be more difficult to assess. This predictive framework has yet to be widely adopted for vector control, although indications are that some species may be more amenable to predicting insecticide resistance phenotypes than others [45,46,47]. In this study, phenotypic assessment of resistance was not possible with frozen samples; hence, we assessed markers of IR. The findings here support the previous literature that suggests *Cx. quinquefasciatus* has a very complex insecticide resistance phenotype, with multiple mechanisms contributing substantially, suggesting that it may be more difficult to predict insecticide resistance to a particular class of pesticides. Therefore, more studies are needed to examine the distribution of both target-site and enzymatic insecticide resistance mechanisms across many populations to determine whether a high-value set of markers can be identified or whether a model can be developed to predict phenotypic insecticide resistance.

In summary, we present baseline insecticide resistance mechanism data for *Cx. quinquefasciatus* populations in Haiti and find that differences exist. Based on the relatively high frequency of pyrethroid insecticide resistance in other phenotypically characterized populations, it is likely that all these Haitian populations are also substantially resistant [29]. We also expect organophosphate resistance to be present but more variable across populations [28]. Although we did not detect important human pathogens in this study, the known human cases of *Culex*-transmitted diseases in Haiti make effective control of *Culex* mosquitoes important, and the insecticide resistance data presented here indicate this may be challenging.

## Figures and Tables

**Table 1:** Primers used for knockdown resistance (*kdr*) and acetylcholinesterase (*AchE*) mutation detection assays and *Culex quinquefasciatus/nigripalpus* (Cxq_n) speciation assay.

Primer Name	Sequence
*kdr*_1014F [13]	TTCACGCTGGAATACTCACGACA
*kdr*_1014L [13]	GGGCGGCGGGCAGGGCGGCGGGGGCGGGGTTCACGCTGGAATACTCACGACTA
*kdr*_1014S [13]	AGCGCGGAGCGCGGTTCACGCTGGAATACTCACGACTG
*kdr*_1014r [13]	GGATCGAATCCATGTGGGACTGCAT
*AchE*_2340S	CCGGCAGGCCGACGGCGACGACTGTGGATCTTCGGGGTTA
*AchE*_2340G	CTGTGGATCTTCGGGGGTG
*AchE*_2362_r	GTGGTCGTACACGTCCAGCG
Cxq/n_Cxq	GCGGGCAGGGCGGCGGGGGCGGGGGGAGCTCCAGATATGGCCTTT
Cxq/n_Cxn	GGAGCTCCTGATATAGCTTTC
Cxq/n_r	ATGAAGGAGGTAGTATTCAAAAACTTAT

## Data Availability

All data supporting this study are found in the manuscript and the associated Supplemental Files. Sequencing data is available from NCBI under BioProject Accession: PRJNA1332129.

## Supplementary Materials

The following supporting information can be downloaded at https://www.mdpi.com/article/10.3390/insects17030331/s1. File S1: Sampling collection metadata, genotyping, and enzymatic activity results for samples used in this study. File S2: Statistical analysis of target site mutations and enzyme assay data. File S3: Raw results of microbiome analysis.

## Abbreviations

The following abbreviations are used in this manuscript:

WNV	West Nile virus
kdr	knock down resistance
AchE	acetylcholinesterase
NFW	nuclease-free water
MCA	melt curve assay
Tm	melting temperature
dscDNA	double-stranded cDNA
Ct	cycle threshold
GST	glutathione S transferase
CytC	cytochrome C oxidase
NT.bpm	NT/NR database matching bases per million
